# Chronic lymphocytic leukaemia during pregnancy: management and thoughts

**DOI:** 10.3332/ecancer.2015.592

**Published:** 2015-11-12

**Authors:** Patricia Winckler, Anna Vanazzi, Maddalena Bozzo, Giovanna Scarfone, Fedro A Peccatori

**Affiliations:** 1 Serviço de Oncologia Médica, Instituto Português de Oncologia de Lisboa, Francisco Gentil, Rua Professor Lima Basto, Lisboa 1700-023, Portugal; 2 Division of Haematology Oncology, European Institute of Oncology, Via Ripamonti 435, Milan 20141, Italy; 3 Department of Obstetrics and Gynaecology, DMCO San Paolo, Via di Rudinì 8, Milan 20142, Italy; 4 Department of Obstetrics, Gynecology and Neonatology, IRCCS Fondazione Cà Granda, Ospedale Maggiore Policlinico, Via della Commenda 12, Milan 20122, Italy; 5 Fertility & Procreation Unit, Division of Gynecologic Oncology, European Institute of Oncology, Via Ripamonti 435, Milan 20141, Italy

**Keywords:** chronic lymphocytic leukaemia, management, pregnancy

## Abstract

Chronic lymphocytic leukaemia (CLL) is the most common leukaemia in the Western world. Despite this fact, its coexistence with pregnancy is extremely rare, with few cases reported in the literature. Given the rarity of this event, it is difficult to conduct large prospective trials to evaluate diagnostic, management, and outcome aspects. The existing evidence is limited to the few published cases and scarce data from reviews on haematological malignancies and pregnancy. Here, we report a case of a 36-year-old patient who had already finished treatment for CLL and was under surveillance when she got pregnant. We describe the evolution of the pregnancy and of the disease’s behaviour as well as the oncological and obstetrical management. Being an indolent disease, CLL during pregnancy can be usually followed up without treatment, but infectious and autoimmune complications might have a significant impact on the pregnancy outcome. Therefore, pregnancy must be closely monitored in specialised centres.

## Introduction

Chronic lymphocytic leukaemia (CLL) is the most common leukaemia in the Western world. Despite this fact, its coexistence with pregnancy is extremely rare, with few cases reported in the literature [1–6]. This is mainly due to the fact that the median age at diagnosis for this disease is 72 years, with only about 10% of patients with CLL being younger than 55 years old [7].

Given the relative rarity of this event, it is difficult to conduct large prospective studies to evaluate diagnosis, management and outcome of CLL during pregnancy. The existing information is limited to reviews on haematological malignancy during pregnancy and to the few published case reports [6–12].

Here, we report the case of a patient who had already been treated for CLL and got pregnant during surveillance. We describe the evolution of the pregnancy and of the disease’s behaviour as well as the management of this coexistence.

## The case

A G2P2 36-year-old female presented to her primary care physician for enlarged cervical and axillary lymph nodes in December 2010. She had no relevant family history nor known comorbidities apart from obesity (BMI: 33.9 kg/m^2^). The two previous pregnancies and deliveries had been uneventful. A routine blood test showed leucocytosis and lymphocytosis (55.340/mcl and 35.417/mcl, respectively), with mild anaemia (haemoglobin 11.5 g/dl). The immunophenotype of the peripheral blood revealed monoclonal B-lymphocytes (CD5+, CD23+) compatible with B-CLL. A fluorescence *in situ* hybridisation (FISH) test showed a chromosome 13 deletion (13DS319). A whole-body computerised tomography (CT) scan confirmed multiple pathological lymph nodes at cervical, mediastinal, axillary, coeliac, hepatic, and retroperitoneal stations. She received rituximab/cladribine combination for 4 cycles followed by maintenance rituximab every 2 months for a total of eight administrations. No significant toxicities were reported and the patient achieved a clinical and radiological complete response, with minimal circulating residual disease. Seventeen months after the end of rituximab, the patient got unintentionally pregnant. Her white blood cell count was 15.350/mcl with 9.970 lymphocytes/mcl, and all the other haematological parameters were within the norm. A cervical, axillary, and inguinal ultrasound was normal and the embryo was well implanted. At 28 weeks gestation, a glucose curve demonstrated gestational diabetes that was treated with diet and subcutaneous insulin. The foetal parameters were normal, without intrauterine growth restriction (IUGR) or macrosomia. At 32 weeks gestation, the patient presented with high fever, shivering, and cough. A thoracic ultrasound performed demonstrated left lobar pneumonia that was successfully treated with intravenous ceftriaxone and clarithromycin. At 34 weeks, the patient developed cholestasis with elevated liver enzymes, hyperbilirubinaemia and pruritus treated with ursodesossicholic acid; at 36+ weeks gestation, steroids were administered in order to increase the foetal pulmonary surfactant, as an amniocentesis performed showed that full foetal maturity was not yet reached. Because of the concomitant presence of cholestasis and suspicion of maternal disease progression (progressive leucocytosis, lymphocytosis, and thrombocytopenia) an increased risk of endouterine death was recognised and anticipation of labour was decided. Therefore, at 37 + 4/7 weeks, labour was induced with cervical prostaglandins and the patient delivered a healthy 3,580 g female baby, with an Apgar score of 10/10 at 1 and 5 min, respectively. There were no obstetric or neonatal postpartum complications. Four months after delivery, her lymphocyte count started to increase and an ultrasound assessment showed several pathological lymph nodes (cervical, axillary, hepatic). She remained under close observation with monthly haematological and clinical examinations. At the time of this report, she is still under surveillance without any treatment.

## Discussion

Managing CLL during pregnancy poses several challenges. At present, few cases have been reported in the literature [1–6], and thus, no guidelines can be used to address clinical questions and any decision should be individualised and tailored to the specific situation. Here, we review different aspects in the light of the existing evidence.

### Contraception and family planning in patients with cancer

Advising on contraception and family planning for patients with cancer is as important as advising on fertility issues. It constitutes a preventive measure in order to avoid difficult situations, including the psychological and physical impact of pregnancy termination, when deemed necessary. It is important that healthcare providers remember to discuss contraception when facing a young patient diagnosed with cancer. A recently published paper showed that of the 29 patients who became pregnant during cancer staging or treatment in a large multi-institutional registry, contraception had been absent in 45% of cases and had failed in 24% of cases [13].

In a review of haematological malignancies in pregnancy [12], the authors recommended avoidance of pregnancy for at least 2 to 3 years in order to confirm a durable remission. CLL was not included in the review, and this disease has the particularity of being incurable; therefore, relapsing is inevitable. The risk of relapse or progression needs to be assessed on an individual basis. Patients who have undergone treatment and are planning a pregnancy need information regarding the risk of disease progression during pregnancy, the possibility to treat the disease during pregnancy and the potential foetal side effects of the proposed treatments [6].

In our case, the pregnancy was not planned and condoms were occasionally used to avoid conception. Nonetheless, when faced with the uncertainties related to the scarce evidence in the literature regarding CLL during pregnancy, the patient was keen to continue with the pregnancy and never considered abortion as an option.

### Concomitant diagnosis of cancer and pregnancy

When cancer is diagnosed during pregnancy or when pregnancy occurs after cancer diagnosis, many issues arise. Independently of the type/stage of the disease, it is important to give the pregnant patient and her family all the available information regarding the different aspects of her disease, including therapeutic alternatives and maternal and foetal risks [14]. In addition to medical indications, emotional condition, beliefs, culture, family dynamics, and financial and legal issues may also affect choice. Each decision should be made together with the patient after thorough information [14]. The decision-making process should involve not only the oncologist and the patient, but also the family. Multidisciplinary cooperation may help to get the full picture and clarify what is the best solution. Besides the oncologist, the obstetrician and the neonatologist, other professionals should participate, including psychologists, social workers, and family counsellors. In cases where major ethical dilemmas arise, the physician’s primary obligation is towards the patient, including autonomy of choice and respect for her values [15].

### Possible impact of cancer treatments on pregnancy outcomes

Rituximab is a FDA (Food and Drug Administration) pregnancy category C drug (risk cannot be ruled out) and is not recommended during pregnancy or lactation. It has the potential of delayed B-cell reconstitution lasting months and occasionally years after administration. Thus, when possible, pregnancy should be avoided during treatment and for 12 months after the last dose of rituximab. Neonatal lymphopenia and/or B-cell depletion were described in case reports of pregnancies exposed to rituximab during the second and third trimesters [16–20]. In a paper reviewing 231 pregnancies associated with maternal rituximab exposure [16], 153 had a known outcome, with 90 live births, 22 of which were premature. One newborn died at 6 weeks of life from unknown causes, even if pregnancy occurred 14 months after the last dose of rituximab; it should be underlined, however, that the pregnancy was complicated by systemic lupus erythematosus, gestational diabetes and exposure to mycophenolate mofetil, warfarin sodium and prednisone. It should also be pointed out that in most of the cases reported in this paper chemotherapy was also given during pregnancy, which constitutes a confounding factor. No long-term studies on B-cell or immune function of children exposed *in utero* have been performed.

Cladribine is a purine analogue with demonstrated clinical activity in CLL [21]. As for all the purine analogues, cladribine reduces the rate of circulating CD4+T lymphocytes, thus inducing a variable immune suppression that could be exacerbated by the pregnant status. It is a category D drug (positive evidence of risk) as teratogenic effects were observed in animal studies. There are no data from human pregnancies. Concerning fludarabine there are also no reports of its use during human pregnancies and it is a category D drug as well. Its antimetabolites are teratogenic and its use during pregnancy should be avoided [8].

### Management of CLL during pregnancy

The indolent nature of CLL allows for delayed therapeutic interventions, and a watchful waiting approach can be adopted in asymptomatic patients [6, 22]. Therefore, therapeutic termination of pregnancy is avoidable [9]. Most patients can be monitored closely without treatment until delivery or disease progression. In case a patient undergoes no treatment, information regarding the risk of disease progression (or of relapse if in remission) and of complications, and concerning the potential need for treatment or supportive measures should be given [6]. Surveillance consists mainly of clinical evaluation and blood tests and does not carry any foetal risk. If need be, ultrasound can be used to evaluate lymph nodes status, with no radiation exposure. In our case, pregnancy occurred when the patient was asymptomatic and not in need of any treatment. In case of serious disease progression and urgent need to treat, the chosen drug would have been controversial. Chlorambucil, though a category D drug, could have been an option, based on case reports of its use in the first and third trimester [5, 8]. As congenital abnormalities have been described with its use in the first trimester, it should preferably be administered after the 15th week of pregnancy [6].

### Interaction between CLL and pregnancy

CLL has a potential impact on pregnancy as it increases the risk of susceptibility to infections, cytopenias and autoimmune phenomena.

Infectious episodes constitute potential serious maternal and foetal risks. It is important to identify and treat all infections in a timely manner and, when possible, take preventive measures [6]. Not all antibiotics can be safely administered during pregnancy, even if penicillin derivatives, macrolides, and cephalosporins have a very low risk of foetal toxicity. In our case, the intravenous combination of cephalosporins and macrolides obtained a complete remission of pneumonia, which was probably due to the concomitant immune depression of gestational diabetes, LLC, and pregnancy. Of particular concern are cytomegalovirus (CMV) and herpes virus (HSV), which are part of the TORCH group (toxoplasma, rubella, CMV, and HSV), because of the increased risk of reactivation in CLL and the potentially catastrophic neonatal complications [6].

Cytopenias may also lead to a severe event, including infection and bleeding. In the setting of marrow failure, erythrocyte and platelet transfusions should be used to maintain a haemoglobin level of ~10 g/dL and a platelet count of >50–100 × 10^9^/L (with close monitoring of platelets nearer the time of delivery) [6].

Moreover, autoimmune phenomena can be observed and they may cause IUGR and metabolic complications. Corticosteroids can be used in this setting [8], when needed and with due caution considering the possible complications of its delivery during pregnancy [6].

One case of gestational diabetes during CLL has been described in the literature [3], but the patient was under corticosteroids. Still, the authors underlined that during pregnancy circulating E-selectin and vascular cell adhesion molecule-1 (VCAM-1) have been found elevated in gestational diabetes. As VCAM-1 levels increase with CLL burden, a possible correlation cannot be ruled out. In our case, the patient’s high BMI was an adjunct risk factor for developing gestational diabetes.

It would also be interesting to understand if the pregnancy could have any impact on the course of the CLL. In the case of our patient, we observed that even though the disease was slowly progressing before pregnancy, it seemed to stabilise during pregnancy, with a slow progression after delivery (Table 1). Nonetheless, a direct causal relation cannot be established from our case.

### Placental invasion and vertical CLL transmission

Not many data are available about placental invasion in haematological malignancies occurring during pregnancy. Defence mechanisms of the placenta in maternal malignancies have not been clarified and vertical transmission of the malignancy is very rare, even if the placenta is involved. If it occurs, it probably depends on homozygosity of the HLA haplotype in mother and foetus, congenital immune deficiency of the foetus, or early transmission of the malignant cells to the immunologically immature foetus [3]. Data on placental invasion in pregnant CLL patients are scarce. Two cases in the literature [1, 4] describe the presence of cells consistent with CLL in the placenta, possibly due to maternal blood contamination. Another case [3] describes that no placental invasion was observed. The clinical significance of these findings is not clear [8].

## Conclusions

Pregnancy occurring during CLL is very rare and there are no specific guidelines. When facing such a situation, one must take several aspects into account. First of all, patient information and education is essential. Before any treatment starts, when facing a young woman diagnosed with cancer, there must be a discussion not only about fertility but also on contraception and family planning. Pregnancy and cancer is a delicate and very specific issue that requires skilled professionals, involvement of the patient and her family and multidisciplinary discussion. Though unpredictable, CLL is usually an indolent disease that allows an observational approach. Still, each case should be examined and independently evaluated regarding the overall conditions. The decision to introduce or delay chemotherapy should be individualised and should be balanced against the impact on maternal and foetal survival and morbidity. It is also important to be aware of previously administered treatments and their potential toxicities as well as to be informed of what treatments can be done in such a situation. Possible complications of CLL might have significant impact on the pregnancy and therefore must be closely monitored. As it is a rare situation, there are still several undefined issues, including the relevance of placental invasion and the existence of any prognostic effects of the pregnancy on the CLL course. Thus, it is essential to report these cases within a well-structured registry like the one organised by the European Society of Gynaecologic Oncology (ESGO) and its working group the International Network on Cancer Infertility and Pregnancy (INCIP).

## Conflicts of interest

The authors report no conflicts of interest.

## Figures and Tables

**Table 1.** Evolution of some of the disease parameters throughout the follow-up.

Time after end of treatment	6 M	9 M	1 Y + 2 M	1 Y + 6 M	1 Y + 8 M	1 Y + 9 M	2 Y	2 Y + 2 M	2 Y+ 3 M
Disease parameters									
**WBC**	N	N	13.250/mcl	16.100/mcl	18.500/mcl	18.880/mcl	20.650/mcl	39.330/mcl	40.480/mcl
**L**	N	N	6.228/mcl	10.620/mcl	12.580/mcl	11.510/mcl	15.070/mcl	21.632/mcl	31.970/mcl
**Hb**	N	N	N	N	N	N	N	N	N
**Plat**	N	N	N	N	N	N	117.000/mcl	128.000/mcl	103.000/mcl
**PBL**	22%	33%	Not performed	Not performed	Not performed	Not performed	Not performed	75%	Not performed
**Sympt**	No	No	No	No	No	No	No	No	No
**LN**	Small ↑	Stable	Small ↑	Stable	Stable	Not performed	Not performed	↑	Not performed

WBC – White Blood Cell count, L – Lymphocytes, Hb – Hemoglobin, Plat – Platelets, PBL – percentage of Pathological B Lymphocytes (immunophenotype analysis of the peripheral blood), Sympt – presence of symptoms, LN – lymph node size (image assessment), N – normal, ↑ – increase, Y – year(s), M – month(s).
